# Efficacy and Students' Perceptions of Virtual Reality in Clinical Education: A Narrative Review

**DOI:** 10.7759/cureus.82852

**Published:** 2025-04-23

**Authors:** Christopher Houser, Alexander Goodman, John Mack, Bradley Simon

**Affiliations:** 1 Simulation, Rocky Vista University College of Osteopathic Medicine, Parker, USA

**Keywords:** augmented reality, clinical decision-making, clinical judgment, clinical reasoning skills, healthcare education, mixed reality, simulation in medical education, virtual reality

## Abstract

Virtual reality (VR) technology has evolved significantly since its inception in the 1950s, now offering advanced, affordable, and immersive experiences that have made it increasingly popular in healthcare education. This narrative review assesses the effectiveness of VR in teaching clinical knowledge, specifically non-technical clinical skills such as clinical reasoning and decision-making, compared to traditional learning methods. The review also evaluates students' perceptions of VR as a learning tool. Our search identified 13 relevant studies, including randomized controlled trials, literature reviews, and a meta-analysis, which predominantly targeted medical, nursing, and physiotherapy students. The results demonstrate that VR often outperforms traditional lecture-based and text-based models in enhancing clinical knowledge and non-technical skills. However, some studies reported mixed or negative outcomes, particularly in areas like patient history-taking and critical thinking skills. Students generally expressed positive attitudes toward VR, citing higher satisfaction, engagement, and motivation compared to traditional learning methods. Despite these promising findings, the review highlights the need for more targeted research, especially in comparing VR with traditional simulation models and evaluating its effectiveness across different healthcare professions. In conclusion, while VR shows great potential in enhancing clinical education, further research is needed to confirm its efficacy and determine its optimal role in medical training.

## Introduction and background

Virtual reality (VR) is a scientific and technical domain that uses computer science and behavioral interfaces to simulate the behavior of three-dimensional (3D) entities in a virtual world, which interact in real time with one or more users in pseudo-natural immersion via sensorimotor channels [1]. In other words, VR is a computer program with a complex user interface allowing subjects to interact with a virtual simulation using real senses such as sight and sound. VR systems were first developed in the 1950s. They included 3D visual, audio, haptic, and olfactory stimuli, but gained little popularity due to their high cost [2]. Today, with advances in technology, VR has become more advanced, affordable, and accessible. Recent advances have introduced features such as head-mounted displays that provide a more immersive VR experience for the subject through clear imaging, quality sound, and fast head tracking. Additionally, newer extended reality platforms, like augmented reality (AR) and mixed reality (MR), are being developed to incorporate elements of the real world into the virtual experience [3].

With these advances in technology, VR has slowly gained popularity in the field of education [2-5]. Simulations now allow for immersion into environments where students can learn about complex concepts in a more engaging manner [2]. VR is also capable of teaching students intricate spatial relationships in fields such as chemistry and human anatomy via a 3D visual interface, and it is believed that VR can lead to longer retention of learned material. Previous research has shown that immersive VR systems can promote better episodic memory performance for users due to the engaging nature of the programs [6]. Additionally, research has demonstrated that VR and MR can improve long-term knowledge retention when compared to traditional learning modalities [7,8].

In recent years, VR has become popular within medical education due to its ability to simulate patient interactions, real-life procedures, and human anatomy [9]. Through VR simulations, students can explore detailed spatial relationships between anatomic structures, interact with virtual patients, order tests, speak with other care team members, and start therapeutic interventions. It is a cost-effective and easily accessible way to prepare students for the clinical portion of their education by giving students clinical experience without leaving the classroom [10]. Students have shown a positive attitude toward the use of VR in their medical education, finding VR learning to be more engaging and enjoyable than traditional methods [11,12]. VR not only enables students to learn course material in an alternative way but also allows them to interact with a virtual simulated patient in a safe, low-stakes environment. For these reasons, a significant amount of research has been conducted on the anatomical, surgical, and psychomotor applications of VR technology within medical education [9,11-17]. While there is a considerable body of research on VR broadly, the role of VR in clinical education remains not entirely understood.

Most of the VR research within medical education has been focused on motor skills and visuospatial anatomy. However, a large portion of pre-clinical medical education is dedicated to clinical medicine and interpersonal skills. In the early years of medical education, it is crucial for students to refine their professional skills to be prepared for clinical training [10]. VR technology provides a unique and valuable opportunity for students to practice these skills in reproducible scenarios and receive helpful feedback. For years, medical schools have utilized the "standardized patient" model, in which live actors act as real patients in controlled and simulated patient encounters [5]. Many institutions have begun to employ VR as a replacement for these standardized patient encounters, given that VR technology has become more cost-effective and streamlined for convenient, repeated use [5]. As VR becomes more commonly integrated in medical curricula, studying the effectiveness of this teaching modality for clinical education will be beneficial. The goal of this literature review is to assess the efficacy of VR in teaching clinical case scenarios, specifically focusing on non-technical clinical skills when compared to traditional teaching models. Additionally, it is essential that students perceive VR as an approachable and effective learning tool; therefore, students’ perceptions of VR in clinical education have been evaluated in comparison to traditional teaching models.

## Review

Methods

Goals of the Literature Review

The primary outcome of this review was to assess the current literature regarding the efficacy of VR in teaching clinical case scenarios compared to traditional lecture-based and text-based models. The endpoints assessed included non-technical clinical skills such as reasoning, decision-making, judgment, critical thinking, and problem-solving ability. The secondary goal was to evaluate students’ perceptions of VR in clinical education when compared to traditional lecture-based and text-based models.

Search Strategy

**Figure 1:**
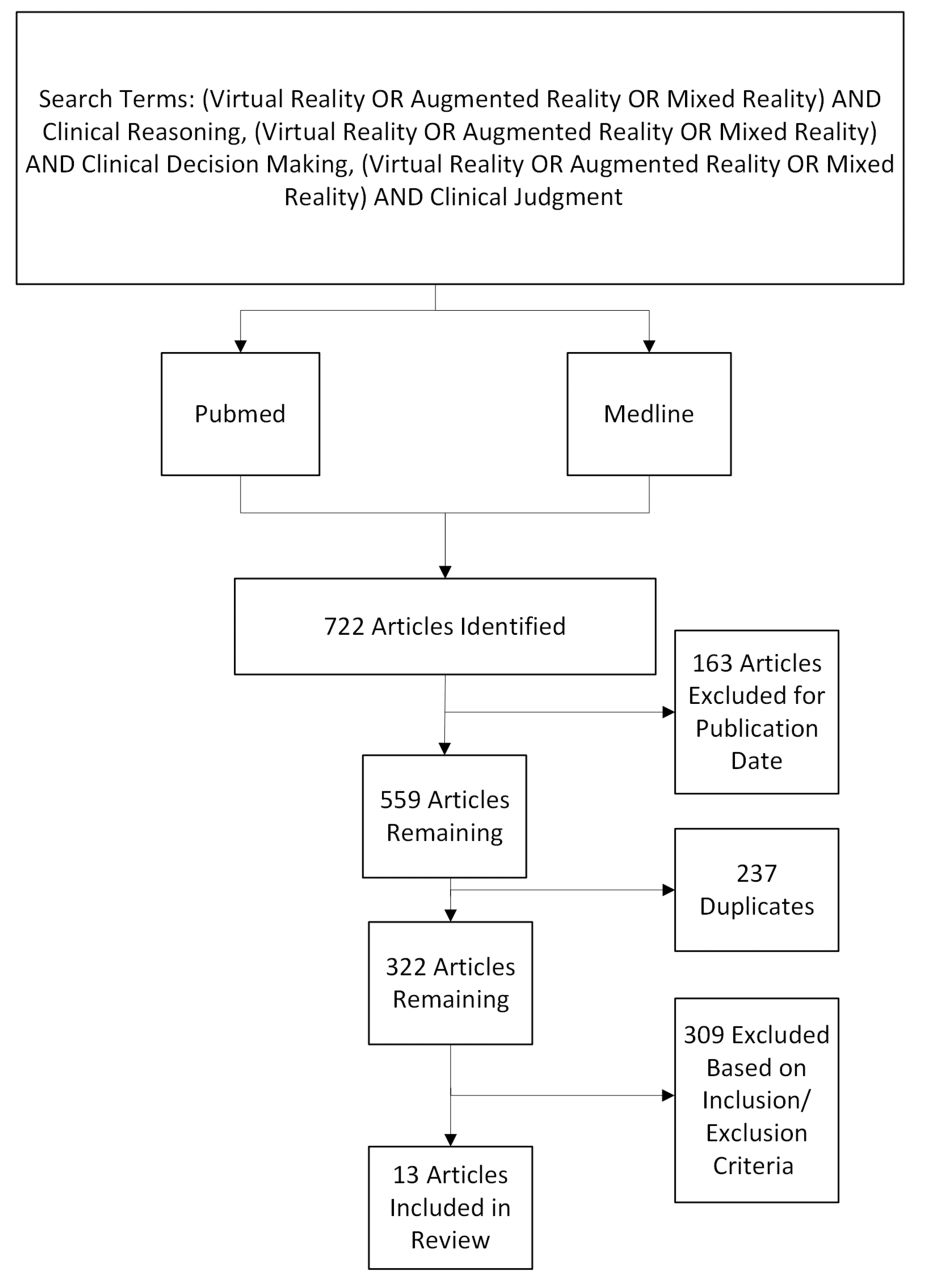
Flow diagram of search results and article selection

Two research databases, Medline and PubMed, were searched for studies investigating VR, AR, and MR in relation to clinical reasoning, critical thinking, clinical decision-making, and clinical judgment. Search terms used were: (Virtual Reality OR Augmented Reality OR Mixed Reality) AND clinical reasoning, (Virtual Reality OR Augmented Reality OR Mixed Reality) AND clinical decision making, (Virtual Reality OR Augmented Reality OR Mixed Reality) AND clinical judgment. Given the rapidly evolving nature of VR technology and research in this field, we established a five-year cut-off for our review, starting in 2023, thereby establishing our date range as 2018-2024. Search criteria and results are shown in Figure 1.

Eligibility Criteria

Inclusion criteria included: a focus on VR, AR, or MR within healthcare education, a focus on healthcare professions students, assessment of either efficacy or student perception, and publication between 2018 and 2024. 

Articles were excluded if published prior to 2018, had no published results, had been retracted, or were unavailable in English due to institutional limitations.

Special focus was given to articles written for medical students, medical residents, physician assistant students, nursing students, and other clinical health professionals requiring clinical reasoning, treatment, or disease management. Following the initial search, over 700 articles were identified. After filtering and removing duplicates, 322 articles were identified for screening. After screening each article and applying inclusion and exclusion criteria, 13 articles were identified that met the inclusion criteria. Notably, one article was excluded because no English translation was available.

Results

Study Characteristics

Out of the 13 articles included in this review, 10 investigated the primary research question. Among these 10 articles, six were randomized controlled trials (RCTs), three were literature reviews, and one was a meta-analysis. The RCTs targeted medical students (two studies), nursing students (three studies), and physiotherapy students (two studies). The articles encompassed diverse populations across multiple countries and included different study designs. Of the 13 studies included in this review, nine assessed students’ perceptions and feelings toward VR in clinical education. The details of all included studies have been outlined in Table 1.

**Table 1:** Summary of included studies in this review VR: Virtual reality; EG: Experimental group; CG: Control group

Study (Authors, Year)	Study Design	Population	Groups (EG, CG)	Outcomes	Results
Dhar et al., 2023 [18]	Scoping review	Medical students	Varied	Decision-making, knowledge retention, and technical clinical skills. The secondary point was satisfaction, usability, and feasibility	One study found improved decision-making competency, five out of six studies showed improved knowledge retention, while one showed no significant difference. All demonstrated high satisfaction with VR.
Mansoory et al., 2021 [19]	Randomized controlled trial	50 medical students	EG=VR-based serious gaming scenario method, CG=Lecture	Improvement in pre-test to post-test knowledge and usability scale	The VR group outperformed the control group in the post-learning exam, and students gave VR high usability ratings.
Watari et al., 2020 [20]	Randomized controlled trial	169 fourth-year medical students	EG=VR simulation group, CG=None	Knowledge, clinical reasoning	Students in EG showed a significant increase in post-test scores on knowledge and clinical reasoning questions.
Padilha et al., 2019 [21]	Randomized controlled trial	42 nursing students	EG=VR, CG=Traditional in-person simulation	Knowledge, clinical reasoning	The VR group had significantly better knowledge retention and clinical reasoning scores, as well as higher satisfaction scores.
Yang and Oh, 2022 [22]	Randomized controlled trial	88 nursing students	EG=VR, CG=In-person simulation and lecture-based material	Knowledge score, problem-solving ability, clinical reasoning, self-confidence	The VR group showed the greatest increase in knowledge scores and problem-solving ability. No difference in clinical reasoning across groups. The VR group had the highest self-confidence scores.
Liu et al., 2023 [23]	Meta-analysis	Nursing students	Varied	Theoretical knowledge, practical skills, and critical thinking	The VR group demonstrated higher theoretical knowledge scores. They found no difference between groups in critical thinking skills.
Jans et al., 2023 [24]	Integrative review	Nursing students	Varied	Knowledge, clinical reasoning, and clinical decision-making	10 of the included studies demonstrated significantly improved knowledge, clinical decision-making, and clinical reasoning in the VR group, while three studies found no difference.
Çetinkaya et al., 2024 [25]	Mixed method	52 physiotherapy students	EG=VR + traditional environment, CG=Traditional learning environment	Achievement scores for clinical reasoning skills, student perception	Similar pre-test scores among both groups, but the VR group performed significantly better on the post-test. Students in the VR group reported high levels of satisfaction with the learning experience.
Bonnin et al., 2023 [26]	Randomized controlled trial	48 physiotherapy students	EG=VR simulation group, CG=Text-based learning group	Patient assessment, diagnosis, and history taking	EG performed significantly worse than CG in patient assessment, history taking, and diagnosis.
Lucena-Anton et al., 2022 [27]	Systematic review	Physiotherapy students	Varied	Clinical decision-making, student perception	Two of the seven studies investigated clinical decision-making skills, and both found no significant difference between groups. Varied results in student perception.
Pal et al., 2020 [28]	Single-arm trial	100 third-year medical students	EG=VR simulation group, CG=None	Students' confidence in non-technical skills after using VR	Students reported increased confidence in non-technical skills.
Yang et al., 2024 [29]	Explanatory sequential mixed methods design	122 nursing students	EG=Integrated virtual simulation, CG=Face-to-face simulation	Self-reported clinical judgment ability, satisfaction, self-confidence	EG significantly improved in self-reported clinical reasoning skills compared to CG. No difference in satisfaction or self-confidence. Qualitatively, the majority of students in EG showed support for VR simulation in improving clinical reasoning skills.
Havola et al., 2021 [30]	Randomized controlled trial	40 nursing students	EG=VR, CG=Computer-based simulation	Simulation game scores, self-reported clinical reasoning skills	Both groups showed an increase in self-reported clinical reasoning scores and game scores.

Efficacy in Teaching Clinical Knowledge

Overall, seven of the 10 studies demonstrated the effectiveness of VR in teaching clinical knowledge, yielding positive results compared to traditional learning models. Many of these studies assessed complex clinical reasoning and diagnostic skills with reassuring outcomes as well. However, three reviewed studies showed equivocal or inferior results compared to traditional models. A large-scale review assessing VR in medical education, completed by Dhar et al., screened more than 2,800 studies across four databases [18]. They eventually included 28 studies in their review, six of which focused specifically on clinical education. Out of these six studies, five found a benefit in either teaching decision-making competency, clinical knowledge, or technical clinical skills. One of the included studies found no significant difference in knowledge between the VR and control groups. Most of the included studies assessed either motor skills, procedural skills, or knowledge, but only one surveyed critical thinking or clinical reasoning.

Medical students: The RCTs targeting medical students showed favorable results for the efficacy of VR. A 2021 study by Mansoory et al. in Iran compared students’ pre-test vs. post-test knowledge following a clinical case scenario of a comatose patient [19]. A total of 50 students were randomized into either a VR treatment group or a lecture-based learning group. They found that students in the VR group performed significantly better than those using lecture-based learning. While both groups improved their scores following the case scenario, the VR group significantly outperformed the lecture group on the post-learning test. Another RCT by Watari et al. conducted in Japan showed that students had improved scores on clinical reasoning and knowledge questions after two VR clinical case scenarios focused on managing patients with altered mental status and acute chest pain [20]. This study included 169 fourth-year medical students across two years. While this study had sufficient power, it lacked a control group to compare the efficacy of VR to traditional models. Nevertheless, the article showed promising results and demonstrated the potential of VR to not only improve knowledge but also enhance clinical reasoning in a clinical case scenario.

Nursing students*: *Two of the RCTs focused specifically on nursing student populations, both of which showed increased efficacy of VR compared to other learning modalities [21,22]. In a 2019 study by Padilha et al. involving 42 nursing students, researchers demonstrated that nursing students using VR had significantly better knowledge scores and knowledge retention compared to a control group in a respiratory distress clinical case scenario [21]. Yang and Oh investigated clinical knowledge scores, clinical reasoning, and problem-solving ability scores in VR against in-person simulation and online lectures in teaching neonatal resuscitation to nursing students [22]. This was a large study with 88 total participants conducted in 2022. Both the VR group and the in-person simulation group showed increased knowledge scores from pre-test to post-test. There was a greater increase in knowledge scores in the VR group, and both the VR and in-person simulation groups showed a significant increase in the scores compared to those in the online lecture group. Additionally, the VR group showed the greatest increase in problem-solving ability scores as well. However, there was no difference in improvement in clinical reasoning among the three groups.

A meta-analysis conducted by Liu et al. in China assessed 12 studies targeting more than 1,100 nursing students [23]. Eleven of the 12 studies investigated theoretical knowledge, and while the results had high heterogeneity, a random effects model revealed that VR technology significantly improved students' theoretical knowledge compared to traditional models. Regarding critical thinking skills, only three of the 12 studies evaluated this endpoint, and a random effects model found no significant difference between the VR and control groups in this area. Of note, one of the included RCTs that demonstrated improved knowledge and clinical reasoning had no control group for comparison with their experimental VR group, and it was unclear if the assessment they utilized was a validated tool. Jans et al. published an integrative review of 18 studies assessing VR’s impact on clinical decision-making among nursing students [24]. Ten studies in this review demonstrated significantly better knowledge and clinical decision-making among the VR groups, while three studies demonstrated no significant difference when compared to controls. The remaining studies did not directly report an outcome from a VR-based intervention. The studies included in the review by Jans et al. tested multiple non-technical clinical skills, including clinical knowledge, clinical decision-making, clinical judgment, and clinical reasoning. The VR groups consistently performed significantly better than the control groups in knowledge questions, clinical decision-making, and clinical reasoning. However, a major limitation is that most of the included studies used subjective questionnaires to assess these dimensions.

Physiotherapy and rehabilitation students: The 2024 study by Çetinkaya et al. in Turkey investigated the efficacy of VR compared to traditional learning models in 52 physiotherapy and rehabilitation students [25]. They found that the VR group performed significantly better on a post-knowledge assessment compared to the control group using more traditional learning methods. However, it is worth noting that the experimental group received both VR and traditional learning tools. Similarly, the RCT in 2023 by Bonnin et al. in France included 48 physiotherapy students completing a clinical case on respiratory insufficiency. They found that the VR group performed significantly worse on the knowledge assessment compared to the text-based learning group, specifically on the history-taking aspects of the assessment. No specific reasoning skill was assessed [26]. Finally, the review by Lucena-Anton et al. in 2022 focused on the efficacy of VR/AR in teaching physiotherapy students compared to traditional models [27]. Two of the seven studies included in their review specifically investigated clinical decision-making skills and found no significant difference between the VR/AR and control groups.

Students' Perceptions of VR

The studies assessing students’ perception of VR as a learning tool consistently found that students rated VR education favorably. Specifically, students enrolled in healthcare education rated the usability, authenticity, and their self-confidence highly after VR learning sessions.

Medical students: In the review by Dhar et al., the authors concluded that medical students were overall satisfied with VR learning opportunities and rated the feasibility and usability highly across multiple studies [18]. Pal et al. investigated a cohort of 100 medical students who perceived the clinical environment to be overwhelming. In their single-arm trial, they found that students were able to increase their confidence and non-technical skills after using a VR simulation [28]. Additionally, focus groups with this same cohort following the VR simulation showed that the students felt they had improved in these areas. Lastly, in the RCT of 50 medical students by Mansoory et al., students gave the VR modality high ratings for user-friendliness despite being unfamiliar with the technology [19].

Nursing students: The RCT conducted by Padilha et al. found that in a group of 42 nursing students, those who used VR for learning had higher satisfaction levels compared to traditional models [21]. Additionally, the RCT by Yang and Oh, which involved 83 nursing students, demonstrated that self-confidence scores of nursing students using VR increased significantly compared to those using in-person simulation and lecture-based learning materials [22]. While the VR, traditional simulation, and lecture-based groups all demonstrated higher confidence scores from pre-test to post-test, the VR group increased their confidence score by an average of 16 points, compared to 6.5 points for the simulation group and 4.4 points for the control group. The same authors also found that the VR and simulation groups had higher learning motivation scores when compared to lecture-based materials among these students. Reviews by Liu et al. and Jans et al. indicated that nursing students using VR reported high levels of satisfaction and motivation following VR learning experiences [23,24]. One of the studies included in the review by Jans et al. demonstrated that students perceived VR as realistic, clinically relevant, and appropriately complex.

An explanatory sequential mixed methods study conducted by Yang et al. compared self-reported assessments of non-technical clinical skills in a group of 122 nursing students, equally divided into two groups. The first group was taught how to manage an intensive care unit (ICU)-specific scenario via face-to-face simulation, while the second group received an integration of VR simulation training in addition to the face-to-face simulation. They found that the students in the integrated group reported significantly greater improvements in both the interpretation of clinical data and the effective discovery of clinical data than the face-to-face simulation group [29]. When assessing overall satisfaction between the two groups, they found no significant difference; however, a majority of the nursing students in the integrated group qualitatively expressed support for the VR simulation [29]. A study by Havola et al. similarly looked at self-reported non-technical skills following the use of computer-based or VR-based simulations in a group of 40 nursing students. They found an increase in students' self-reported clinical reasoning skills following the use of both computer-based and VR-based simulations, which positively correlated with their simulation scores. However, nursing students engaged more with the VR simulation than the computer simulation [30]. 

Physiotherapy and rehabilitation students: Çetinkaya et al. reported that the physiotherapy students tested were satisfied with VR learning after their experiences [25]. However, the authors did not comment on the satisfaction of the control group, making it difficult to determine if there was a difference in perception between the two teaching modalities. The RCT by Bonnin et al. found that 15 of 23 physiotherapy students believed that VR would be useful in their future education, and 17 of those same 23 students found it to be more motivating than traditional models [26]. Finally, in the review by Lucena-Anton et al., five of the seven included studies measured students' learning satisfaction using VR/AR platforms. One study found significantly improved learning satisfaction, another found significantly improved engagement and enjoyment, another found improved self-efficacy, one reported better emotional perception among VR-based learning groups but less learning satisfaction, and the last study found no difference in students' perception or experience [27].

Discussion

The focus of this narrative review was to assess the effectiveness of VR in clinical education, particularly regarding clinical knowledge and non-technical clinical skills. The secondary goal was to assess students’ perception and attitude toward the use of VR in medical education.

Efficacy in Teaching Clinical Scenarios

Generally, the literature indicates that students who utilized VR for clinical cases showed greater clinical knowledge and non-technical skills than their peers. Specifically, VR demonstrated its potential to improve clinical knowledge in seven studies [20-25]. As for non-technical clinical skills, VR improved clinical reasoning in three studies [20,22,24], decision-making in two studies [18,24], clinical judgment in one study [24], and problem-solving ability in one study [22]. A majority of the articles included in this review comparing VR to traditional learning modalities found that students using VR outperformed those using text-based learning, lecture-based learning, and in-person simulation in multiple areas involving clinical knowledge and non-technical skills. These results reinforce the potential benefit of using VR in clinical education. 

However, several studies showed mixed results or outright negative findings. In the RCT conducted by Bonnin et al., physiotherapy students using VR performed significantly worse on the knowledge assessment compared to the text-based learning group [26]. Specifically, the students using VR performed worse on patient history taking but showed equal performance on diagnosis. This calls into question whether specific information presented in VR is more difficult for students to retain or recall compared to when reading it from text [31]. VR relies heavily on students’ auditory and visual senses and requires them to interpret and encode sensory information in real time, similar to what is required in real patient interactions [5]. In three different literature reviews, researchers identified studies where VR demonstrated no significant difference compared to traditional learning modalities. In two reviews, several studies were identified that showed no significant difference in clinical knowledge when comparing VR groups to control groups [18,24]. The meta-analysis by Liu et al. demonstrated that VR had no significant difference in critical thinking skills compared to traditional education modalities [23]. Furthermore, the review by Lucena-Anton et al. found no significant difference in clinical decision-making skills between VR groups and controls [27]. This inconsistency of results between studies, as well as the limited number of available studies, suggests that further research is needed to determine the true effectiveness of VR in medical education.

Students' Perceptions

Regarding students’ perception of VR, the included studies found that most students had positive feelings toward VR education. In general, students felt VR prepared them well for future assessments, found it engaging, and were more motivated to use VR than other traditional learning methods. Multiple studies found that students had higher satisfaction when using VR and believed that it would be useful in their future education [19,20]. These results were not surprising considering the advancements in VR technology in recent years. As VR technology has improved, so has its ability to replicate real patient encounters, making it much more useful and engaging. Our findings of student perception agree with much of the current research regarding VR in education, in that healthcare students perceive VR as more satisfactory than traditional learning modalities [19-28]. Overall, the included studies demonstrated that students from multiple fields (medicine, nursing, rehabilitation) rated VR learning very positively and found it realistic. They found VR significantly more engaging and satisfying to use than other traditional modalities such as lectures and in-person simulation. Students' positive attitudes toward VR in medical education may facilitate its integration into healthcare curricula in the years to come. Even despite conflicting evidence on the efficacy of VR in teaching non-technical skills and promoting knowledge retention, it is likely that the application of VR within medical education will continue to grow and broaden, given the high satisfaction levels reported by students [5].

Limitations

The included studies were heterogeneous in their populations, methods, measurement techniques, and results. It was difficult to compare studies that were vastly different, especially those that measured different outcomes. Many of the studies used unique VR programs and cases, further complicating the comparison between results. No differentiation was made between varying healthcare professions (i.e., medical versus nursing versus other) for the purpose of this review. Clinical skills can differ significantly between groups of healthcare professions, so reviews on more targeted populations would be useful. Our literature search revealed studies focusing on medical, nursing, and physiotherapy students, but other professions, like physician assistant students and pharmacy students, need to be evaluated as well before VR can be integrated broadly into healthcare education.

Most studies did not include their specific assessment tools or elaborate on the types of questions students were asked during their assessments. Given this, it was impossible to determine how much assessments differed between studies. Additionally, establishing objective assessments of clinical reasoning, clinical decision-making, and clinical judgment is very challenging. Attempting to assess these parameters subjectively can give certain learning models unfair advantages due to bias. Each student is unique and has different study preferences, making this topic especially challenging to quantify and analyze.

Future Directions

In future studies, we suggest a more targeted approach to reviewing this literature. The application of VR in education is extremely broad, and assessing its utility may require a focused approach, such as concentrating on a specific healthcare profession, more specific clinical cases, and validated assessment tools. Additionally, while VR is being utilized for the education of multiple healthcare professions, it is important to differentiate these populations when assessing its efficacy because educational goals may differ between the curricula of these professions. Clinical reasoning and decision-making are important in both nursing and medicine, but each profession's approach to clinical problems differs. Furthermore, much less research is focused on VR compared to traditional simulation models, such as those involving patient actors. Traditional simulation models remain the predominant educational tool in medical education, and future studies should investigate the efficacy of VR compared to these models.

## Conclusions

Overall, we found compelling evidence for the use of VR in teaching clinical cases among healthcare professionals. In multiple reviews and RCTs, students using VR outperformed control groups using traditional educational tools, such as lecture-based and text-based modalities. Several studies concluded that students using VR performed significantly better on knowledge assessments than those using traditional modalities. Furthermore, more than one study found that students using VR excelled in assessments of clinical reasoning, clinical judgement, decision-making, critical thinking, and problem-solving. While more than one study showed no benefit of VR in improving knowledge or critical thinking skills compared to traditional education modalities, these represent a smaller subset of the included studies. Students of all types consistently chose VR modalities as the more exciting, engaging, and preferable medium for learning. This is not to be discounted, as student enjoyment and satisfaction are vital aspects of education. Further research is necessary to determine the usefulness of VR within clinical education, but the current findings show great promise in teaching not only clinical knowledge but also non-technical clinical skills.
